# Unraveling the Contact Network Patterns between Commercial Turkey Operation in North Carolina and the Distribution of *Salmonella* Species

**DOI:** 10.3390/pathogens10121539

**Published:** 2021-11-25

**Authors:** Cameron Ellington, Claude Hebron, Rocio Crespo, Gustavo Machado

**Affiliations:** 1 Department of Population Health and Pathobiology, College of Veterinary Medicine, Raleigh, NC 27607, USA; cahinso2@ncsu.edu (C.E.); rcrespo@ncsu.edu (R.C.); 2 Prestage Farms, Inc., Clinton, NC 28328, USA; chebron@prestagefarms.com

**Keywords:** *Salmonella*, network, risk factors, food-borne, turkey, North Carolina, prevalence

## Abstract

Salmonellosis originating from poultry poses a significant threat to human health. Surveillance within production is thus needed to minimize risk. The objectives of this work were to investigate the distribution of *Salmonella* spp. from a commercial turkey operation and describe the animal movement patterns to investigate the association between contact network structure and *Salmonella* infection status. Four years of routine growout farm samples along with data on facility location, time since barns were built, production style, and bird movement data were utilized. From all of the surveillance samples collected, *Salmonella* serotyping was performed on positive samples and results showed that the most represented groups were C1 (28.67%), B (24.37%) and C2 (17.13%). The serovar Infantis (26.44%) was the most highly represented, followed by Senftenberg (12.76%) and Albany (10.93%). Results illustrated the seasonality of *Salmonella* presence with a higher number of positive samples being collected in the second half of each calendar year. We also demonstrated that *Salmonella* was more likely to occur in samples from older farms compared to farms built more recently. The contact network connectivity was low, although a few highly connected farms were identified. Results of the contact network showed that the farms which tested positive for *Salmonella* were not clustered within the network, suggesting that even though *Salmonella* dissemination occurs via transferring infected birds, for this study case it is unlikely the most important route of transmission. In conclusion, this study identified seasonality of *Salmonella* with significantly more cases in the second half of each year and also uncovered the role of between-farm movement of birds as not a major mode of *Salmonella* transmission.

## 1. Introduction

*Salmonella* infections in poultry are a health risk to the birds and later on to humans as a foodborne pathogen. The consumption of turkey in the U.S. has increased 17-fold since 1909 [1]. Scharff estimates *Salmonella* species in poultry to cost approximately $2.8 billion annually in the U. S. [2]. Consumption of undercooked contaminated products can cause acute, severe gastroenteritis in humans [3]. This straight, non-spore-forming Gram-negative rod can be quite challenging to control as the introduction to and maintenance within poultry operations is multifaceted. More than 2500 serovars exist within the subspecies *enterica* with about 10% of those being found in poultry production [3]. Groups are designated based on the somatic (O) and flagellar (H) antigens [4].

Adult turkeys are often asymptomatic with *Salmonella* infections but can transmit the bacteria through eggs to poults [5]. Vaccination is available and often implemented in breeder turkey flocks as a means to control certain serovars that pose a higher risk to human health. A 2014 study utilizing data from another commercial production company in the US showed that 90% of turkey farms that sampled positive for *Salmonella* via drag/bootie swabs tested positive for *Salmonella* at the processing plant [6], emphasizing the need to identify and mediate this pathogen prior to the production of a product that is destined for human consumption.

Incidences of *Salmonella* in turkey flocks has been estimated at between 16% and 54% [5]. The introduction of *Salmonella* to the flock can occur from many sources since it is a bacterium transmitted both horizontally and vertically (internal and/or external contamination of eggs) [5]. From the hatchery to the processing plant, *Salmonella* is a constant threat to poultry production systems. The main modes of transmission include contaminated feed, biologic vectors (birds, rats, mice, mites, insects), contaminated water and mechanical vectors/fomites (trucks, personnel, equipment) [5]. Transmission may occur between farms and between houses on a farm when on-farm biosecurity (use of personal protective equipment including bouffant, clean coveralls, clean boots or boot cover and gloves, disinfection of vehicle prior crossing established clean-dirty line of property, cleaning and disinfection of equipment used within multiple houses during visit, use of boot disinfection stations between entering houses, and following of all company protocols for personal visits to farms) is not being followed, as shown by several other studies that analyze the movement of other diseases such as avian influenza through the use of a network model [7,8]. Current literature specifically on the analysis of the movement networks and the dissemination of *Salmonella* within turkey production is limited. In the present study, a contact network was created by following the movement of turkey flocks from their brooding location to their growout location. Following these pairs would enable the identification of brooder farm locations that were more or less likely to repeatedly test positive for *Salmonella* at the surveillance sampling time point.

The transportation of live turkeys to the processing plant is the final opportunity to expose the birds to *Salmonella* once they leave a growout facility [9]. Proper cleaning and disinfection of transport coops is essential to ensure the minimal transmission of bacteria. The ability of *Salmonella* to persist in products deemed for human consumption has forced processing plants to consider decontamination treatments as the easiest way to control this pathogen during processing [3].

Multiple studies have shown the value of using contact networks paired with between-flock movement of personnel to implement improved surveillance and intervention to combat disease [10,11]. However, there is limited information on network analysis [7,10,11,12,13,14,15] and disease transmission models in poultry systems [7,10]. Furthermore, no information is available in the literature on the transmission of *Salmonella* among turkey flocks. In this study, we described the distribution of *Salmonella* among the sites of one turkey producing company located in North Carolina and the spread along the movement of birds from the brooder to the growout farms’ network. This work is based on *Salmonella* cases collected approximately two weeks prior to harvest from growout farms from 2017 to 2020. Additionally, we assessed the distribution by serotype and group.

## 2. Results

### 2.1. General Description of the Collected Database and the Distribution of Salmonella spp. Groups and Serotypes

**Figure 1:**
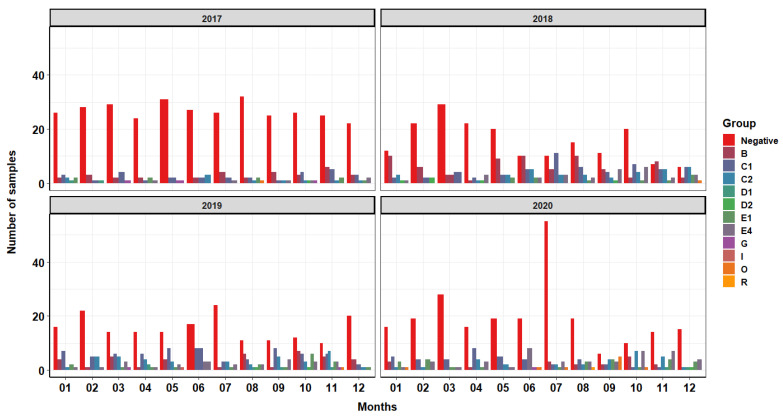
Distribution of *Salmonella* groups over time. This figure represents the monthly incidence of *Salmonella* groups from bootie swab samples collected from growout turkey farms approximately two weeks prior to harvest from 2017 to 2020. These figures include samples collected from both conventional and antibiotic-free reared turkeys. More information can be found in Supplementary Table S1.

**Figure 2:**
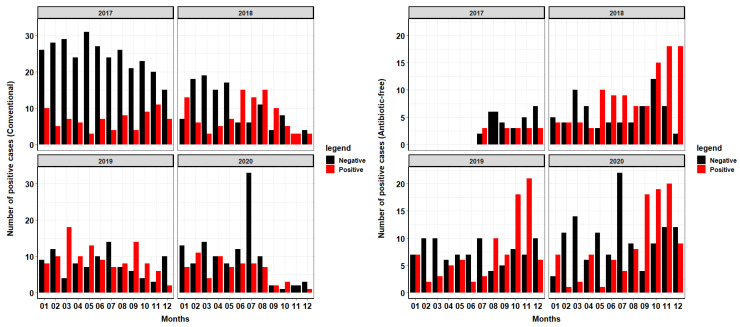
Prevalence of *Salmonella* in conventional vs. antibiotic-free rearing systems over time. This figure shows the prevalence of *Salmonella* from samples collected via bootie swabs at growout farm approximately two weeks prior to harvest from 2017–2020. More information can be found in Supplementary Table S1.

In this study, we considered an operation composed of 50 brooder and 76 growout turkey farms that range in age from 1–30 years and are geographically spread throughout 8 counties in the state of North Carolina. The following tables and figures show results from routine surveillance samples collected from growout farms approximately two weeks prior to harvest. North Carolina as a state is second, behind Minnesota, when it comes to the number of turkeys produced annually. The production system considered in this study has protocols that parallel many others, making it an ideal system to analyze and provide conclusions which apply to other systems in similar geographic areas. Positive samples from bootie swabs (i.e., “shoe cover swabs” as defined by the National Poultry Improvement Plan Program Standards Section 3(a)(1)(iii)) consisted of 30 separate *Salmonella* serovars encompassing 11 different groups. Figure 1 and Figure 2 show the quantification of collected samples and their proportion within the total number of collected positive samples. The group with the highest frequency of collection was C1 (Table 1). The most frequently collected serovar was Infantis, representing 26.44% of all collected positive samples. *Salmonella* Infantis falls within the group C1 (Table 2).

Our results demonstrate an increased occurrence of group C1, containing the serovar Infantis, through 2018 and 2019 but with a reduction in 2020 compared to previous years (Figure 1). Across all four years, there were more positive *Salmonella* samples in the second half of each year compared to the first half of each year. For more information on serotype case distribution (rather than group) over time, see the Supplementary Materials (Supplemental Figures S1 and S2). Positive samples falling within group B were the second most represented, with a large portion of those samples being collected in the year 2018.

This production system experienced an increase in the collection of positive *Salmonella* samples from flocks reared via antibiotic-free (ABF) style over time (Figure 2). There are also more positive *Salmonella* samples collected from antibiotic-free reared flocks in the second half of each calendar year. By 2020, the majority of all samples collected from September to December are from ABF flocks. Similar trends can be seen in 2018, 2019 and 2020, where from September to November half or more of the samples collected tested positive for *Salmonella* from ABF-reared flocks. The total number of surveillance samples collected from conventional flocks was 981, with 362 (37%) of those samples testing positive for Salmonella. In ABF production flocks, 622 total surveillance samples were collected, with 315 (51%) of those samples testing positive for Salmonella.

#### Facility Age Influence on *Salmonella*

Company data on the year each facility was built was used to determine the farm or facility’s age. Age ranges included greater than 30 years, 20–30 years and less than 20 years. The likelihood of a farm having a positive *Salmonella* surveillance sample increased with the age of the farm, with positive and significant correlation rho = 0.29 (*p* value < 0.005). Farms built 30 years ago or more had a 57% chance of collecting a positive sample; farms built between 20–30 years ago have 54% chance of collecting a positive sample; farms less than 20 years of age have a 29% chance of collecting a positive sample.

### 2.2. Between-Farm Contact Networks

The total number of unique between-farm movements were 358 transported groups, with a maximum out-degree of 28 and maximum in-degree of 14. As expected, the betweenness of this poultry network was zero, which meant there were no intermediate farms from brooder to grower, thus only direct pairs of movements were recorded. Data on the number of nodes, edges and network parameters of connectivity and cohesiveness (GSCC and GWCC) for the entire period (2017–2020) are shown in Table 3. The proportion of the size of GWCC included all farms in the network, and because only direct movements from brooder to growers were recorded, the GSCC was one.

**Figure 3:**
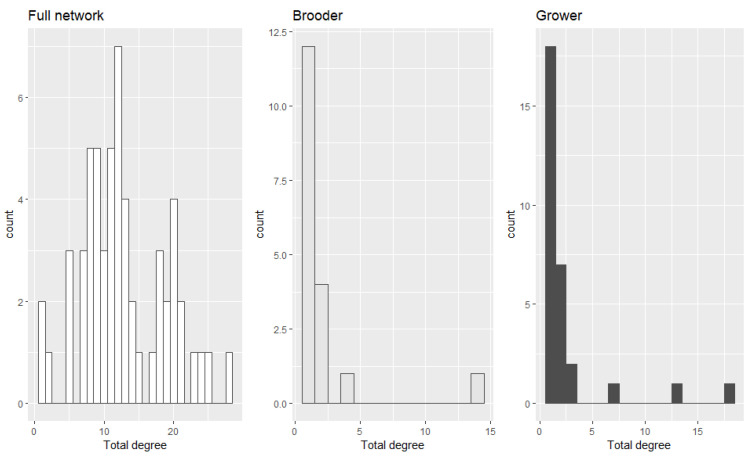
The total degree distribution of the entire network along with the degree distribution of brooders and growers.

In addition, we evaluated the degree distribution of the entire network while also considering the number of contacts of each farm type (Figure 3). Please see Supplementary Material (Figure S3) for the description of in and out-degree of each farm type.

#### The Contact Network for the Movements Involving Infected Movements between Brooder and Growout Farms

**Figure 4:**
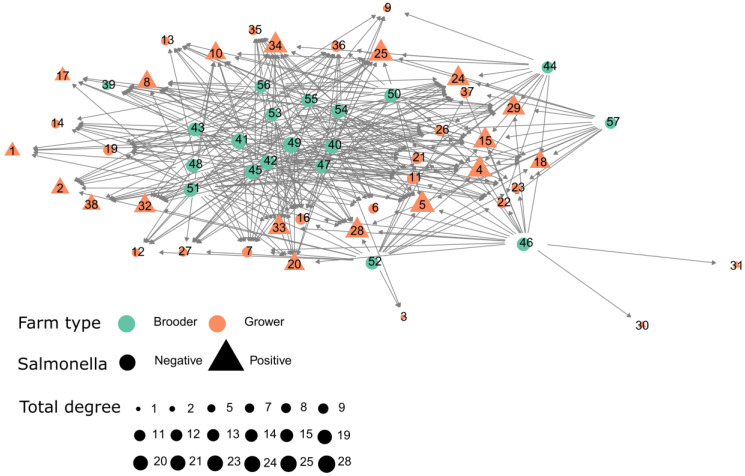
Contact network of farms classified as brooder and grower along with its *Salmonella* status (at least one positive sample within the study period).

The *Salmonella* positive degree, in-degree and out-degree was not significantly different (*p* > 0.05) when compared with the entire contact network, in which mean total degree was 9.08, in-degree and out-degree of 4.54, while the whole contact network exhibits a total degree of 12.63, in-degree and out-degree of 6.31. This suggests that the distribution of *Salmonella* among farms is not compartmentalized between infected and non-infected movement flows, though examining the direct links between brooder and growers reveals that there is substantial mixing of birds at growers coming from brooders that were identified as infected and uninfected (Figure 4). Additionally, we have also run the *K-test* over the infected (at least one positive *Salmonella* result). The direct association between bird movement and *Salmonella* was not significant based on the k-test (*p*-value > 0.05), therefore, farms were not significantly more connected than expected by chance (randomly). This suggests that the contact network does not provide evidence that *Salmonella* is spreading by the movement of infected birds.

## 3. Discussion

The purpose of this study was to identify contributing factors to the spread of *Salmonella* within an eastern U.S. turkey production system by analyzing the prevalence of positive samples collected during routine surveillance of growout flocks and between-farm bird movements in mediation of *Salmonella* dissemination. While the human-health impact of Salmonella is important, it was not evaluated in this study, as information on the impact of working in poultry production on each employee was not available. Rather, the focus was placed on evaluating the incidence and potential movements of this pathogen within this system. In this study, factors that were considered included rearing style (antibiotic-free vs. conventional) over time, impact of brooder location on growout location and age of the farm according to company documentation. Rearing style and seasonality had varying trends and the older a facility was the more likely it was to be positive for *Salmonella* on surveillance sampling in this system. Group C1 was the serogroup with the highest prevalence of *Salmonella*, with almost one third of all samples collected falling into this group, including the most frequently isolated serovar: Infantis. We have also demonstrated that the turkey farms in the evaluated production company are weakly connected through the movement of bird’s brooders directly into growouts. The *Salmonella* infected network showed a lower degree when compared with the entire network, additionally the cases of *Salmonella* did now show a clear association between movement of birds and positive growout sites. Despite the fact that, for *Salmonella,* the infected networks were similar to the non-infected network, we remark that the use of contact networks could become a useful tool to assess the movement-associated risk of other poultry diseases such as avian influenza and Mycoplasmosis. Infected networks were not more infected than farms in the whole contact network.

A 2010 study analyzing *Salmonella* serotypes isolated from turkey cuts at different steps of processing at a processing plant identified 83% (101/122) of their isolates were *Salmonella* Derby, rendering it the overwhelming majority [16] from samples collected. In the present study, we demonstrated that the same serotypes were found in the live production stage, however at much lower rates, i.e., *Salmonella* Derby accounted for less than 1% (0.29%) of the positive samples collected. This suggests that certain serovars may be more likely to be harbored and introduced to incoming carcasses at the processing level rather than in live production. In comparison to broiler live production and processing, the most commonly isolated group is C3, especially *Salmonella* Kentucky [17]. The present study considering live production of turkeys did not have any positive samples from group C3. While there are many serovars of *Salmonella* that affect both species, trends from these surveillance samples indicate that some may be more species-specific than others, including those that fall into group C3 being better adapted to chickens.

We found clear patterns in the occurrence of *Salmonella* in ABF production with peaks in the second half of each calendar year. In contrast, a previous study, also from North Carolina, determined no association between *Salmonella* and seasonality [18] while we found trends in the occurrence of *Salmonella* with peaks from September to November in ABF production as seen in Figure 1 and Figure 2. Analysis of Supplemental Figure S2 shows peaks of positive samples in the months of October and November in years 2018, 2019 and 2020. The entrance of biological vectors into the poultry house is more likely to occur during this time of the year as these animals attempt to evade the cooler temperatures by seeking refuge in the climate-controlled poultry house that also has a readily available supply of food and water. This system determines rearing style (conventional vs. ABF) of each flock at each location based on market demands so this study is unable to assess *Salmonella* prevalence solely based on the rearing style of each location, as each location grows turkeys under both styles. Figure 2 shows an overall increase in the collected positive samples over time from ABF farms over the course of the four years. This trend was consistent with the goals of this production system, as the national demand for ABF turkey at the level of processing has increased over the four years analyzed. In the years 2018–2020 during the months of September through December, many more positive samples were collected from ABF flocks compared to conventional flocks. Conventionally reared flocks appear to show a decline in negative samples collected over the course of the four years, with peaks of positive samples collected inconsistently dispersed throughout the year with occasional peaks in summer months, especially during the years 2018 and 2019. During the summer, naturally ventilated, curtain-sided facilities will often utilize a fogger system to aid in cooling of the birds. These cooling systems can increase relative humidity in the environment 4–5% [19], facilitating the creation of the ideal environment for bacteria growth [20]. In summating all four years of production, this system has a higher prevalence of *Salmonella* in ABF production flock compared to conventional production flock of over 10%, indicating that the use of some antimicrobials may aid in the reduction in *Salmonella* even though therapy may not be targeted specifically at this pathogen.

Since *Salmonella* can be horizontally transmitted within and between flocks, a flock transmitting the bacteria at the time of transfer would continue to pass it from bird to bird after arrival at the growout facility [5]. The ability of a location to harbor a pathogen, specifically *Salmonella*, was considered in this study by identifying flocks sampled at multiple growout locations that all stemmed from the same brooder location. Through our analysis we determined that the presence of *Salmonella* at each growout location is independent of the brooder from which the flock originated within this operation. The brooder farms do not appear to be a source of spread for *Salmonella*. Similarly, the proximity of one location of a farm to another did not appear to be a risk factor for testing positive for *Salmonella*. *Salmonella* was widely distributed across eight counties in which this system grows birds with clusters of farms in the same geographical area not necessarily consistently testing positive for the same serovars confirming that other methods of transmission (such as biologic vectors, fomites, contaminated feed) are a more important source of transmission than geographic proximity with airborne transmission via contaminated dust.

A study in a laying hen system determined that the older the infrastructure of the housing system, the more likely they were to test positive for *Salmonella* [21]. Similar results were found in this study consisting of all curtain-sided, naturally ventilated houses. This result is likely due to better overall infrastructure of newer houses with cleaner and modern construction materials that are designed with good structural biosecurity in mind. It would be expected that all curtain-sided, naturally ventilated houses have more physical openings compared to solid-sided, tunnel-ventilated houses, and consequently a higher chance for the introduction of pathogens via biologic vectors. Additionally, older houses are more likely to have structural deterioration that allows the entrance of biological vectors of *Salmonella* including birds and rodents. It can be concluded that farm age is a risk factor for *Salmonella* presence.

For the North America poultry industry, our results from the between-farm movement suggested that even though there were insignificant associations between movements and *Salmonella* positive cases, key network centrality metrics such as degree could be considered in order to implement risk-based disease surveillance. The utilization of the ranked farms by total degree could be used to disrupt the contact network between infected and uninfected movement flows in the case of *Salmonella* and also in the case of a foreign animal disease (FAD) epidemic [7,8,10]. On the other hand, the extremely low level of clustering of the infected network compared to a random network is suggestive that *Salmonella* might not maintain itself and spread within the poultry network. Therefore, other unmeasured or unknown transmission routes such as proximity of mortality disposal (incineration, on-farm composting) to houses, feed truck time at each facility and movement of company personnel should be further investigated while targeting between-farm *Salmonella* control.

In an ideal study of a poultry production system in which *Salmonella* prevalence and risk factors were being assessed, the management method of rearing turkeys (ABF vs. conventional) would be set by location in order to allow for an analysis of each type of style’s influence on the likelihood of *Salmonella* presence. Similarly, our study focused on surveillance data from growout locations only. One study found the bacteria persist in the dust of a depopulated poultry facility up to one year after the birds were removed [22]. Further work could build on the present study by collecting samples at the brooder level in order to determine if there is true maintenance and/or movement of the bacteria from one location to the next when the flock transfers at five weeks of age within this particular system. The lack of data collection from the brooder location is a limitation of this study as we cannot determine the origin of the bacteria in each flock. Moreover, a consideration of sampling the growout houses multiple times, to investigate whether there is “replacement” of *Salmonella* serotypes over time would be ideal. It would also be beneficial to consider data from breeder farms in an effort to identify any vertical transmission of *Salmonella* within the system. Future work should also include feed truck movement data along with information on movement of service and maintenance personnel. Litter age and management should be included as well. *Salmonella* is a complex and ever-present pathogen within poultry production. While many factors exist that may be contributing to the movement of *Salmonella* within poultry flocks, an analysis of these factors to identify the major risk factors can be accomplished.

## 4. Materials and Methods

### 4.1. General Description of Data and Mathematical Calculations Used to Aid in Data Interpretation

Description of Production System: Poults are placed at day of age on brooder farms typically comprising two houses with a total capacity of 10,500–16,000 poults total. At approximately five weeks of age, birds are transferred from their brooder farm to a growout farm in order to facilitate the expected change in size of each bird through the growing period. Growout farms are typically composed of between 3 and 6 houses with a total capacity of 16,000–21,000 turkeys total, depending on the sex being reared (heavy hens vs. light hens, vs. heavy toms). The growout facility will house the birds until harvest age. At transfer, either an entire flock is moved from a brooder location to a growout location, or two brooder locations are moved to the same growout location to fulfill capacity as explained above. All hen farms are equipped with an incinerator to dispose of dead birds while the tom farms have composting capability. All tom turkeys are raised within the conventional style (reared without the constraints of having to maintain antibiotic-free status) while heavy and light hens are reared either conventional-style or antibiotic-free (ABF) depending on market demands. During the life of the flock, on-farm movements of personnel frequently occur. Feed trucks deliver rations to each farm approximately once each week and perform deliveries to approximately four farms each day. Service personnel assess farms once or twice weekly for growout and brooder age turkeys, respectively. Downtime between flocks on farms varies from 7 to 21 days. Breeder flocks from which hatched poults originate are vaccinated six (toms) to eleven (hens) times prior to going into production with a *Salmonella* vaccine (Poulvac^®^ ST, Zoetis, Parsippany-Troy Hills, NJ, USA).

Sample Collection: Routine samples collected via “bootie swabs’’ are acquired and analyzed approximately two weeks prior to harvest from growout farms. An individual farm consists between three and six houses. For routine collection, all houses at each location are sampled, then samples are pooled together for submission. To perform a “bootie swab” (i.e., “shoe cover swabs” as defined by the National Poultry Improvement Plan Program Standards Section 3(a)(1)(iii)) a plastic shoe bootie is applied to one of both shoes. After walking through the house, the plastic bootie is removed and submitted for analysis in a whirl pak bag.

Sample analysis: Collected samples are submitted to a third-party lab for analysis and serotyping. Traditional benchwork testing is performed using a series of cultures and overnight incubation periods to initially identify positive and negative samples, then differentiate positive samples into serogroups. Lastly, serotyping is performed on positive samples to define the serovar.

Facility age calculation: The total number of times each farm tested positive on surveillance sampling was divided by the total number of samples collected from each farm then multiplied by 100 to calculate the percent chance a farm would test positive. Age was determined using company records and divided into the following categories: old = greater than 30 years, middle = between 20 and 30 years, and new = less than 20 years. In addition, we have evaluated the correlation (Spearman’s rank correlation) between tested negative farms and each facility age.

Descriptive analysis: Table 1 and Table 2 were created by determining the total number of times a particular group/serovar was represented (total number of positive) in data containing all collected samples. The total number of positives for each group/serovar was then divided by the total number of positives within the entire collection to calculate a proportion for each serovar/group. Figure 1 and Figure 2 show the prevalence, or number of positive samples collected according to historical data provided by the company, of *Salmonella* over the course of the four years. Figure 1 shows each group represented by a different color and displayed according to the frequency of collection. Figure 2 shows the prevalence of *Salmonella* over four years according to the production style, antibiotic-free (ABF) and conventional. Quantification of the number of samples collected by each rearing style is shown by the two different colors of the bars within the figure.

Description of nomenclature: *Salmonella* groups are defined using the Kauffmann-White scheme, which uses serologic identification of the O (somatic) and H (flagellar) antigens to assign groups then further separates the organism into serovars [4].

### 4.2. Contacts and Networks

#### 4.2.1. Between Farm Contact Networks

To analyze the between-farm contacts, we reconstructed a directed static network. To apply a static network analysis, repeated between-farm contacts between two farms throughout the observation period, from August 2016 to December 2020 were aggregated into a single one. Here, directed ‘edges’ are represented by bird shipments between two ‘nodes’, where nodes correspond to turkey farms. For the static and the time-series networks, we calculated several centrality indicators for farms within the network: out-degree, in-degree, closeness centrality, betweenness, giant weakly connected component (GWCC) and giant strongly connected component (GSCC) (Table 4) [23].

#### 4.2.2. The Contact Network for the Movements Involving Infected Movements

A transmission network is typically a subset of the complete contact network, as not all contacts lead to disease transmission [25]. Therefore, we considered the infected movements as a subdivision of the full network because we were interested in the disease dynamics and how this information can be directly translated into action in the field. The infected network included 27 farms. The network is represented in Figure 4, where infected and uninfected farms are color-coded, while also presenting each farm type. To evaluate whether there was a significant relation between the observed network and the distribution of *Salmonella*-positive cases across the network we performed a path-based k-test, described elsewhere [26]. The *Salmonella*-positive farms were labeled as infected if at least one positive diagnostic was reported. We ran the path-based k-tests [26] with 10,000 iterations. These analyses assessed whether the mean network path length between infected farms was smaller than expected by chance, and therefore the extent to which the social network predicted the occurrence of *Salmonella* cases. All analysis and visualization were performed using R statistical software [27] version (3.4.2).

## 5. Conclusions

In conclusion, this study shows some seasonality of *Salmonella* surveillance samples from growout farms. There appears to be an increase in positive samples collected during the second half of each year. Rearing style does appear to influence this as the results of collected samples as more positive samples were collected from farms utilizing an ABF production style compared to a conventional production style. The movement of birds from brooder farm to growout farm did not have any effect on the likelihood of testing positive for *Salmonella* on surveillance bootie swabs prior to harvest. The array of samples collected is widely distributed across the network with no indication that a single brooder location is contributing to the spread of *Salmonella* to multiple growout locations. Lastly, the factor that seems to have the largest effect on the likelihood of testing positive for *Salmonella* in this system is farm age, with farms less than 20 years of age more likely to test negative compared to farms 20–30 years of age and farms 30+ years of age. Turkey movement data analysis results presented here will be helpful in informing the identification of farms to be targeted as part of *Salmonella* control and surveillance activities. Given the characteristics of the network, directing surveillance, awareness, and biosecurity enforcement targeted at sites with top degree may increase the number of positive samples but is less likely to reduce between farm transmissions.

## Figures and Tables

**Table 1:** Table shows percentages of prevalence distribution for each group.

Group	Number of Positive	Proportion (%)
C1	194	28.67
B	165	24.37
C2	116	17.13
E4	101	14.92
E1	63	9.31
O	12	1.77
D1, D2	9	1.33
G	6	0.89
I, R	1	0.15

**Table 2:** Table shows percentages of prevalence distribution for each serotype.

Serovar	Group	Number of Positive	Proportion (%)
Infantis	C1	179	26.44
Senftenberg	E4	86	12.76
Albany	C2	74	10.93
Schwarzengrund	B	59	8.71
Uganda	E1	56	8.27
Agona	B	40	5.91
Muenchen	C2	28	4.13
1,4,5,12	B	26	3.84
Typhimurium	B	17	2.51
Liverpool	E4	16	2.36
Reading	B	15	2.21
Hadar	C2	10	1.47
Berta, Ouakam, Rissen	*	9	1.33
Alachua	O	8	1.18
Anatum	E1	6	0.88
Lillie, Worthington	*	5	0.74
Heidelberg, Rough O	*	4	0.59
6,7:r:-, Derby, Kiambu	*	2	0.29
16:d:-, 6,8:z10:-, Arizoniae, Johannesburg, Kentucky, Newport	*	1	0.15

* Beta (D1); Oaukam (D2); Rissen (C1); Lillie (C1); Worthington (G); Heidelberg (B); Rough O (O); 6,7:r:- (C1), Derby (B), Kiambu (B); 16:d:- (I); 6,8:z10:- (C2); Arizoniae (G); Johannesburg (R); Kentucky (C2); Newport (C2).

**Table 3:** Network descriptive statistics for all movements from November 2017 to November 2020 of one poultry company in North Carolina.

Parameter	Network Metric at Farm Level
Nodes	56
Edges	358
Sum of moved batches	888
Mean degree	12.78
Centralization	0.14
Max value of in degree	14
Max value of out-degree	28
Max size of GWCC	56 (100%)
Max size of GSCC	1 (1.78%)
Mean betweenness	0

**Table 4:** Description of network analysis terminology and metrics.

Parameter	Definition	Reference
Nodes	The unit of interest in network analysis. For example, premises or slaughterhouses.	[24]
Edge	The link between two nodes in a network.	[24]
Degree (k)	A number of unique contacts to and from a specific premise. When the directionality is considered, the ingoing and outgoing contacts are defined: out-degree is the number of contacts originating from a specific premise, and in-degree is the number of contacts coming into a specific premise.	[24]
Movements	The number of movements as batches are recorded over a certain period of time.	[24]
Betweenness	The extent to which a node lies on a path connecting other pairs of nodes, defined by the number of geodesics (shortest paths) going through a node.	[24]
Giant weakly connected component (GWCC)	The proportion of nodes that are connected in the largest component when directionality of movement is ignored.	[24]
Giant strongly connected component (GSCC)	The proportion of the nodes that are connected in the largest component when directionality of movement is considered.	[24]

## Data Availability

The data that support the findings of this study are not publicly available and are protected by confidential agreements, therefore, are not available.

## Supplementary Materials

The following are available online at https://www.mdpi.com/article/10.3390/pathogens10121539/s1, Figure S1: Distribution of *Salmonella* serotypes overtime, Figure S2: Distribution of *Salmonella* serotypes over times in antibiotic-free and conventional rearing styles, Figure S3: In and out-degree distribution of brooder and growout farms, Data S1: Supplementary data for figures.
