# Supplementary material for: Unraveling the Contact Network Patterns between Commercial Turkey Operation in North Carolina and the Distribution of Salmonella Species
Source: Pathogens. 2021 Nov 25;10(12):1539. doi: 10.3390/pathogens10121539 (PMC8708296; doi:10.3390/pathogens10121539)
Supplement: Supplementary file 1 [file pathogens-10-01539-s001.zip › pathogens-1450591 Supplementary Update.pdf]

5. Supplementary Materials

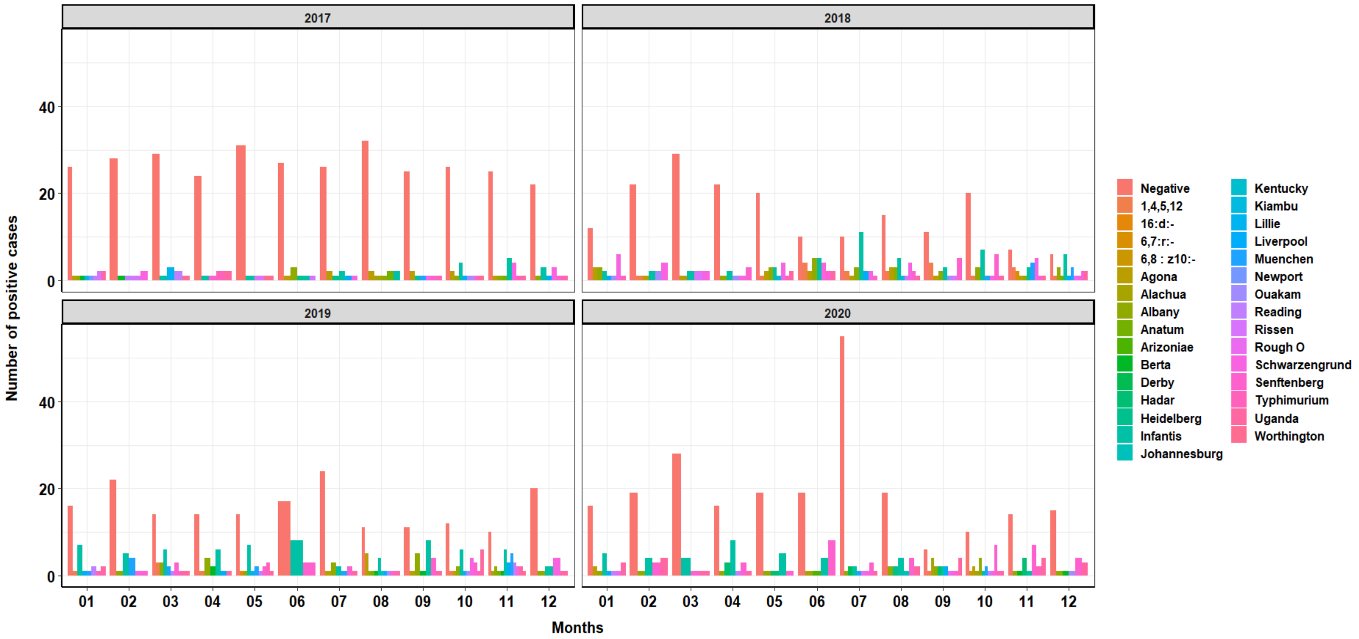

Supplemental Figure 1. Distribution of Salmonella serotypes over time. This figure represents the monthly incidence of Salmonella serotypes from bootie swab samples collected from growout turkey farms approximately two weeks prior to harvest from 2017 to 2020. This figure includes samples collected from boh conventional and antibiotic-free reared turkeys

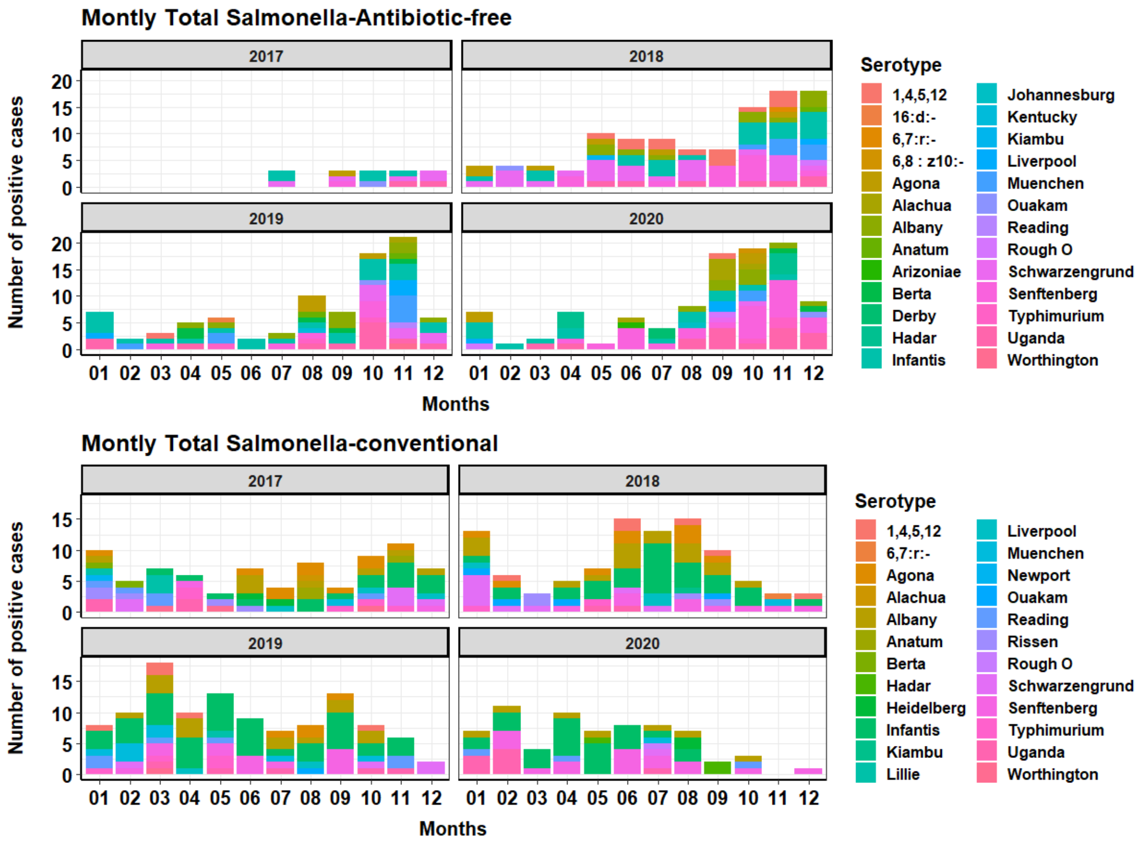

Supplemental Figure 2. Distibution of Salmonella serotypes over time in antibiotic-free and conventional rearing styles. This figure represents the monthly incidence of Salmonella serotypes from bootie swab samples collected from growout turkey farms approximately two weeks prior to harvest from 2017 to 2020.

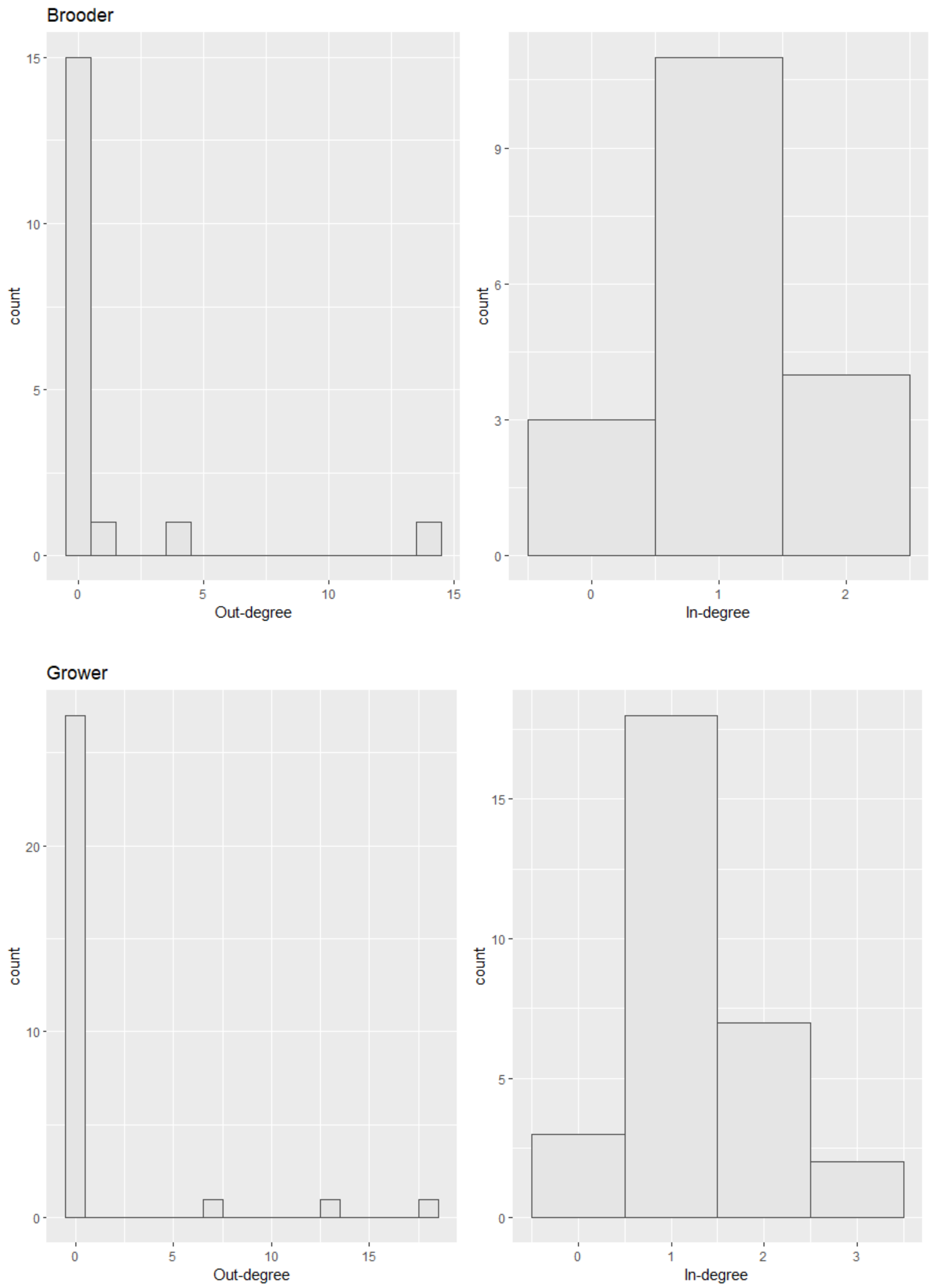

Supplemental Figure 3. In and out-degree distribution of brooder and growout farms.
